# The TRIP technique: A trilumen catheter-assisted contralateral gate precannulation method for distal bifurcated component during thoracoabdominal branched endovascular repair

**DOI:** 10.1016/j.jvscit.2026.102401

**Published:** 2026-07-03

**Authors:** Dai Yamanouchi, Yusuke Sakurai, Masaru Nemoto, Sohei Matsuura, Ryohei Maeno, Shinnosuke Tomioka

**Affiliations:** a Department of Vascular Surgery, Fujita Health University, Toyoake, Aichi, Japan; b Division of Vascular Surgery, University of Wisconsin, Madison, WI

**Keywords:** Thoracoabdominal aortic aneurysm, Branched endovascular aneurysm repair, Endoleak

## Abstract

Contralateral gate cannulation of the distal bifurcated component during thoracoabdominal branched endovascular repair can be challenging in patients with previous endovascular aneurysm repair (EVAR) because multiple indwelling and newly implanted stent graft components may crowd the distal aortic field and obscure wire behavior. We describe a trilumen catheter-assisted contralateral gate precannulation (TRIP) technique for distal bifurcated component during thoracoabdominal branched endovascular repair. A 81-year-old man presented 7 years after infrarenal EVAR with a type IA endoleak from a previous suprarenal fixation stent graft. Because of his operative risk, off the shelf endovascular thoracoabdominal branched endovascular repair was selected. After establishment of through-and-through access, placement of the aortic component, and completion of visceral branch stenting, attention was turned to distal bifurcated component deployment using the TRIP technique. Before deployment of the distal bifurcated component, a trilumen catheter was advanced from the upper-extremity sheath to the ipsilateral femoral sheath over the existing through-and-through wire. A 0.014-inch wire was introduced through the trilumen catheter and passed through the removable guidewire tube of the distal bifurcated component to precannulate the contralateral gate. After deployment of the distal bifurcated component, a 7F, 75-cm-long sheath was advanced over the precannulated wire through the contralateral gate. A 0.035-inch buddy wire was then introduced, snared from the contralateral femoral access, and exchanged to establish secure through-and-through access for contralateral limb delivery. The TRIP technique enabled confident contralateral gate access despite a crowded distal aortic configuration and may be useful during thoracoabdominal branched endovascular repair for type IA endoleak after previous EVAR when conventional gate cannulation is expected to be difficult.

Endovascular reintervention after failed infrarenal endovascular aneurysm repair (EVAR) is increasingly encountered, and fenestrated or branched endovascular repair has been reported as a feasible approach for failed EVAR with proximal seal failure or type IA endoleak.^1^, ^2^, ^3^ However, redo endovascular anatomy can be technically challenging. In particular, patients undergoing thoracoabdominal branched repair for type IA endoleak after previous EVAR may have a crowded distal aortic field due to the previous stent graft, suprarenal fixation stents, newly implanted branched aortic component, visceral branch stents, wires, sheaths, and delivery systems.

The GORE EXCLUDER thoracoabdominal branch endoprosthesis (TAMBE) device (W. L. Gore & Associates) consists of an aortic component, four visceral branch components, a distal bifurcated component, and contralateral leg components. The distal bifurcated component bifurcates from the aortic component and facilitates distal extension into the iliac arteries.^4^ Contralateral gate cannulation of the distal bifurcated component is generally performed after partial deployment of the component. In standard instructions, after the distal bifurcated component is positioned and partially deployed, the contralateral leg hole is cannulated and the contralateral leg component is advanced and deployed. In redo EVAR cases with multiple overlapping stent graft components, conventional retrograde gate cannulation can be difficult because wire movement may be hard to interpret fluoroscopically and inadvertent passage outside the intended gate may not be immediately apparent. Preloaded or precannulated gate strategies have been described in standard EVAR to facilitate rapid contralateral gate cannulation in challenging anatomy.^5^

We describe the TRIP technique for distal bifurcated component during thoracoabdominal branched endovascular repair. This technique uses a trilumen catheter and the removable guidewire tube (RGT) of the distal bifurcated component to establish secure gate access before deployment, allowing confident contralateral limb delivery after deployment.

## Case report

Written informed consent for publication of this case report and any accompanying images was obtained from the patient.

An 81-year-old man with hypertension, hyperlipidemia, chronic obstructive pulmonary disease, and a history of smoking had previously undergone EVAR for an infrarenal abdominal aortic aneurysm using a Medtronic Endurant stent graft with suprarenal fixation. Seven years later, surveillance imaging demonstrated a type IA endoleak. The diameter of the renal artery was 19.3 mm, and the distance between superior mesenteric artery to higher renal artery (*left*) was 4.2 mm. Given his comorbidities and elevated risk for open conversion, thoracoabdominal branched endovascular repair was offered. Currently, no approved fenestrated aortic stent graft is available in Japan. As a result, the recently approved TAMBE and physician-modified endografts remain the only available endovascular options for selected complex aortic cases.

**Fig 1:**
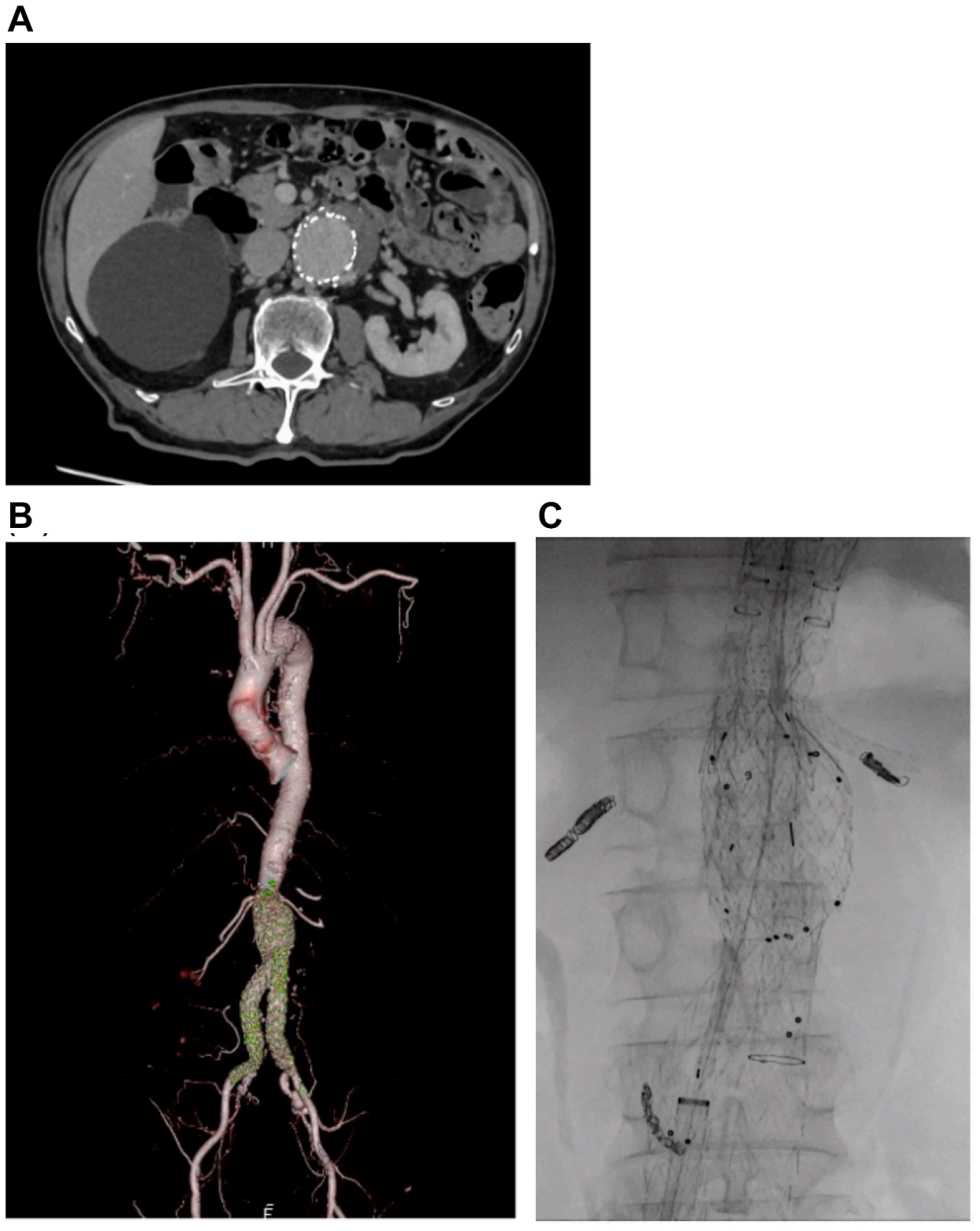
Pre- and intraoperative images. **A,** Computed tomography (CT) image of preoperative type IA endoleak. Preoperative CT angiography demonstrating type IA endoleak. **B,** Three-dimensional (3D) reconstruction of preoperative CT angiogram. Previous infrarenal endovascular aneurysm repair with suprarenal fixation. **C,** Intraoperative image after distal bifurcated component (DBC) full deployment showing congested operative field.

Preoperative imaging demonstrated anatomy suitable for thoracoabdominal branched endovascular repair. Because of the previously implanted infrarenal stent graft with suprarenal fixation and anticipated crowding of the distal aortic lumen, difficulty with standard contralateral gate cannulation of the distal bifurcated component was anticipated (Fig 1, *A* and *B*). A planned TRIP technique was therefore used to facilitate distal bifurcated component gate access.

The procedure was performed under general anesthesia. Right high brachial artery cutdown was performed, and a 12F sheath was introduced. Right common femoral percutaneous access was obtained for the ipsilateral side using a 22F sheath, and left common femoral access was obtained using a 12F sheath for the contralateral side. The bilateral accessory renal arteries were then coil embolized. The inferior mesenteric artery had been coil embolized during the previous EVAR procedure. Through-and-through access was established between the upper extremity and ipsilateral femoral access. TAMBE aortic component (37 × 20 × 160 mm) was advanced and positioned in standard fashion. After partial deployment, the visceral target vessels were sequentially cannulated using a stent-as-you-go technique. The superior mesenteric, celiac, right renal, and left renal arteries were bridged with 7 × 79, 7 × 59, 5 × 79, and 5 × 79 mm balloon-expandable covered stents, respectively. The aortic component was then fully deployed. At this stage, the preexisting EVAR graft, the aortic component of the multibranch endograft, and multiple wires and catheters occupied the same operative field, making accurate cannulation of the contralateral gate of the distal bifurcated component (23-14.5-100 mm) extremely challenging (Fig 1, *C*).

## TRIP technique

After completion of the visceral branch stenting, attention was turned to deployment of the distal bifurcated component. In the standard off the shelf TAMBE procedure, the distal bifurcated component is introduced into the distal end of the aortic component, partially deployed, and the contralateral leg gate is then cannulated. The manufacturer's instructions for use also notes that precannulation of the distal bifurcated component RGT is not required for the standard TAMBE procedure and that the RGT should be removed before introduction into the sheath. In this case, because conventional gate cannulation was expected to be difficult, we used the TRIP technique as a technical modification.

Steps for TRIP techniques:

1. A trilumen catheter was advanced from the upper-extremity sheath to the ipsilateral femoral sheath over the preexisting through-and-through wire (Fig 2, *A*).

**Fig 2:**
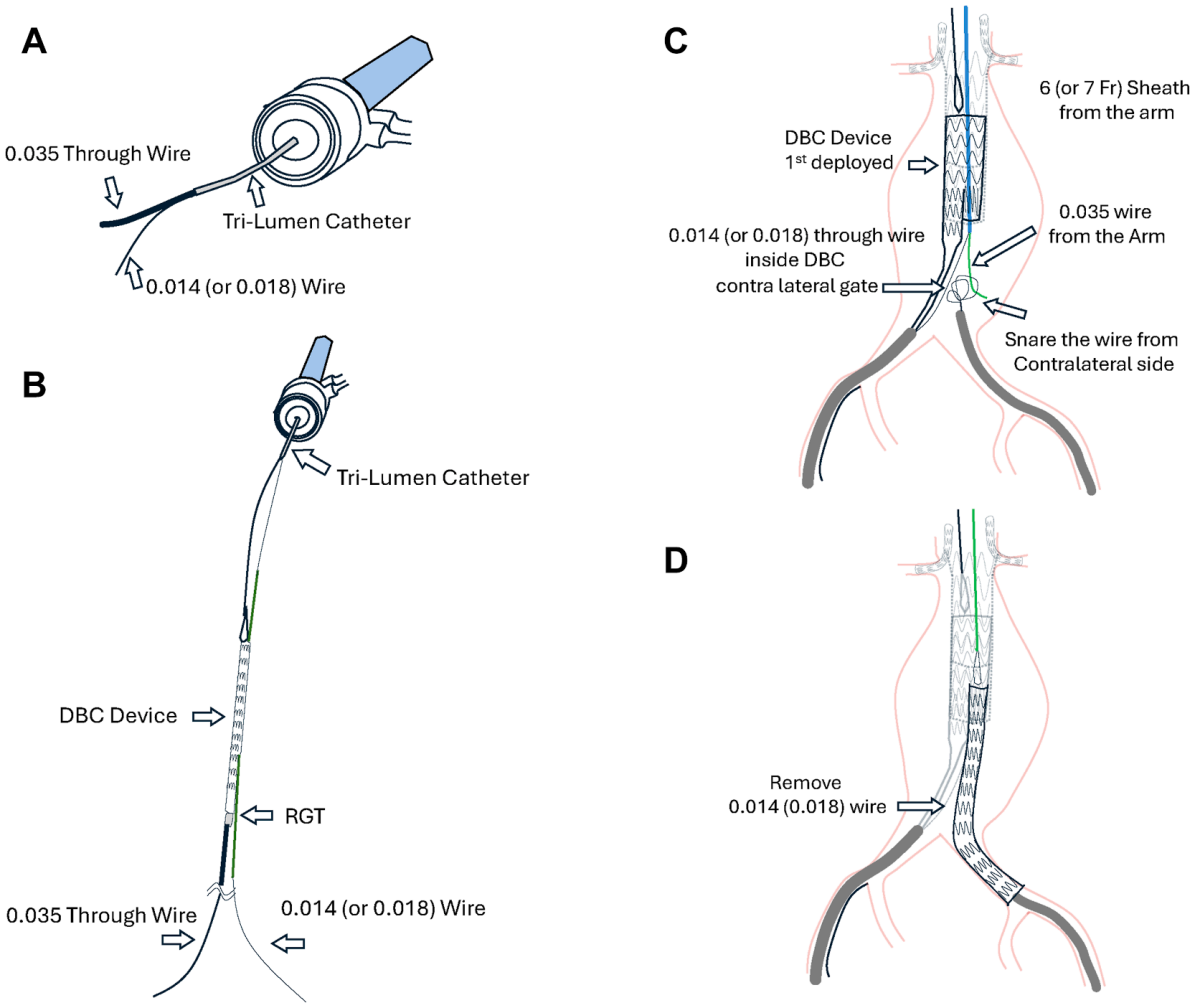
Schema of a trilumen catheter-assisted contralateral gate precannulation (TRIP) technique. **A,** Arm side: Schema of the TRIP technique. A trilumen catheter is advanced from the upper-extremity sheath to the ipsilateral femoral sheath over the preexisting through-and-through wire. **B,** Femoral side: A 0.014- or 0.018-inch wire is then advanced through a separate lumen and passed through the removable guidewire tube of the distal bifurcated component (DBC) to precannulate the contralateral gate. **C,** DBC deployment and snare: After deployment of the DBC, a 6F or 7F long sheath is advanced over the precannulated wire through the contralateral gate. A 0.035-inch buddy wire is introduced through the sheath and snared from the contralateral femoral access to establish through-and-through access. **D,** Contralateral leg deployment: After removal of the 0.014- or 0.018-inch wire, the contralateral leg component is advanced over the snared 0.035-inch wire and deployed in the usual fashion.
2. A 0.014-inch guidewire was advanced through a separate lumen of the trilumen catheter. Using the RGT of the distal bifurcated component, this wire was passed through the contralateral gate before placement and deployment of the distal bifurcated component. This established predeployment gate access (Fig 2, *B*).
3. The distal bifurcated component (23-14-10 mm) was advanced into the distal aortic component and positioned appropriately.
4. The distal bifurcated component was deployed (Fig 2, *C*). After deployment, a 7F, 75 cm long sheath was advanced from the arm side over the precannulated 0.014-inch wire through the contralateral gate of the distal bifurcated component. The sheath tip was positioned either within or just distal to the contralateral gate to secure access.
5. A 0.035-inch guidewire was advanced as a buddy wire through the long sheath. The 0.035-inch wire was then snared from the contralateral femoral access sheath, establishing secure through-and-through access from the arm side across the contralateral gate to the contralateral femoral sheath (Fig 2, *C*).
6. The contralateral limb was advanced over the established wire access and deployed in the usual fashion (Fig 2, *D*).
7. Completion angiography demonstrated patent visceral branches, patent iliac limbs, and exclusion of the type IA endoleak without evidence of type III endoleak (Fig 3).

**Fig 3:**
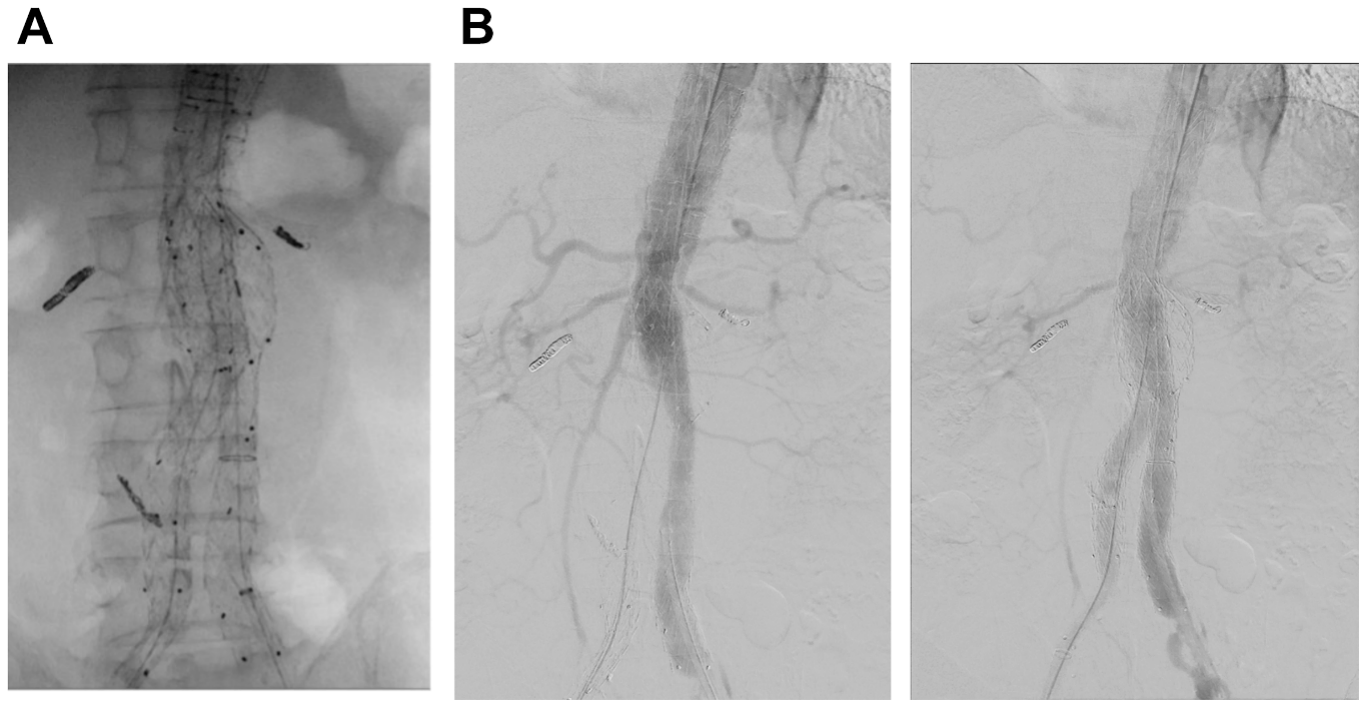
Completion angiogram. **A**, Completion X ray image showing thoracoabdominal branch endoprosthesis (TAMBE) aortic component, branch stent grafts, distal bifurcated component, and iliac extension limbs with previous endovascular aneurysm repair (EVAR) devices. **B,** Completion angiography demonstrating exclusion of the type Ia endoleak with patent visceral branches and iliac limbs.

The total operative time was 222 minutes, fluoroscopy time was 100 minutes, and radiation dose was 1282 mGy.

## Discussion

This report describes the TRIP technique for distal bifurcated component during thoracoabdominal branched endovascular repair. The technique was developed to address a specific technical challenge: difficult contralateral gate cannulation in a crowded distal aortic field after previous EVAR.

For failed infrarenal EVAR, fenestrated and branched endovascular repair has been reported with high technical success and acceptable early mortality in selected patients, supporting its role as an alternative to open conversion in anatomically suitable high-risk.^1^^,^^3^ A prospective multicenter cohort specifically evaluating fenestrated and branched repair after failed EVAR with type IA endoleak also supports this strategy in appropriately selected patients.^2^

In standard thoracoabdominal branched endovascular repair, the distal bifurcated component is introduced after deployment of the aortic component and visceral branch stents. The contralateral gate is cannulated after partial deployment, and the contralateral leg component is then advanced and deployed. This sequence is straightforward in many patients. However, when the indication is treatment of a type IA endoleak after previous infrarenal EVAR, the distal aorta may contain multiple overlapping metallic structures. These may include the previous main body, suprarenal fixation stents, the distal end of the newly implanted thoracoabdominal aortic component, branch stents, multiple wires, and large-bore sheaths. This configuration can make it difficult to determine whether a wire has entered the true contralateral gate, passed between stent struts, entered the space between overlapping grafts, or tracked outside the intended lumen.

The TRIP technique offers several potential advantages. First, the contralateral gate is accessed before deployment, when the RGT provides a direct route through the intended gate. Second, after deployment, access is maintained by the precannulated 0.014- or 0.018-inch wire, reducing the need for repeated attempts at retrograde cannulation. Third, advancing a 6F or 7F long sheath over the precannulated wire provides a stable platform for introduction of a 0.035-inch buddy wire. Finally, snaring the 0.035-inch wire from the contralateral femoral access converts the initial small-wire access into robust through-and-through access suitable for contralateral limb delivery.

The concept of preloaded or precannulated gate access has precedent in standard EVAR. Mirza et al^5^ described preloaded contralateral gate techniques to facilitate rapid gate cannulation in challenging anatomy, including use of a “snare ride” technique. The TRIP technique differs from these previous methods because it is applied to the distal bifurcated component during thoracoabdominal branched endovascular repair and uses an upper-extremity trilumen catheter to precannulate the distal bifurcated component gate through the RGT before deployment.

This technique may be particularly useful in redo EVAR anatomy, narrow distal aortic lumens, severe stent crowding, previous suprarenal fixation, or situations in which fluoroscopic assessment of wire course is limited. It may also reduce procedure time, radiation exposure, contrast use, and wire manipulation in selected cases, although these potential benefits require further evaluation in a larger series.

Several technical considerations are important. The precannulated wire must be carefully maintained during device advancement and deployment. Excessive tension should be avoided to prevent distortion of the distal bifurcated component or displacement of the delivery system. Because the standard instructions for use states that distal bifurcated component RGT precannulation is not needed for the standard procedure and warns that failure to remove the RGT before sheath introduction could result in embolization leading to lower-limb ischemia, this technique should be considered an advanced technical modification for selected complex cases. It should be performed only by operators experienced with branched endovascular repair and the device platform. The TRIP method is that it is currently device-specific. The technique relies on the RGT of the distal bifurcated component to provide a defined path for predeployment contralateral gate access. Therefore, it should not be considered universally applicable to all bifurcated or branched endograft systems.

The present report has limitations. It describes a single case, and no comparison can be made with standard contralateral gate cannulation. The technique also requires upper-extremity access, a trilumen catheter, careful wire management, and familiarity with the distal bifurcated component delivery system. Additional experience is needed to define the ideal indications, safety considerations, and reproducibility of the TRIP technique.

## Conclusions

The TRIP technique is a trilumen catheter-assisted contralateral gate precannulation method for distal bifurcated component during thoracoabdominal branched endovascular repair. In a patient undergoing treatment of a type IA endoleak after previous EVAR, this technique enabled confident contralateral gate access despite a crowded distal aortic configuration. The TRIP technique may be a useful adjunct in selected complex branched endovascular repairs when conventional distal bifurcated component gate cannulation is expected to be challenging.

*The opinions or views expressed in this commentary are those of the authors and do not necessarily reflect the opinions or recommendations of the Journal of Vascular Surgery Cases and Innovative Techniques or the Society for Vascular Surgery.*

## Funding

None.

## Disclosures

D.Y. serves as a consultant to Shape Memory Medical, W.L. Gore & Associates. Y.S., M.N., S.M., R.M., and S.T. declare that they have no competing interests.
